# Microsatellite allele dose and configuration establishment (MADCE): an integrated approach for genetic studies in allopolyploids

**DOI:** 10.1186/1471-2229-12-25

**Published:** 2012-02-17

**Authors:** Thijs van Dijk, Yolanda Noordijk, Tiphaine Dubos, Marco CAM Bink, Bert J Meulenbroek, Richard GF Visser, Eric van de Weg

**Affiliations:** 1 Wageningen UR Plant Breeding, Wageningen University and Research Centre, PO Box 386, 6700 AJ Wageningen, The Netherlands; 2 Graduate School of Experimental Plant Sciences, Wageningen, the Netherlands; 3 Biometris, Wageningen University and Research Centre, PO Box 100, 6700AC Wageningen, The Netherlands; 4 Fresh Forward Breeding B.V. Wielseweg 38a, 4024 BK Eck en Wiel, the Netherlands

## Abstract

**Background:**

Genetic studies in allopolyploid plants are challenging because of the presence of similar sub-genomes, which leads to multiple alleles and complex segregation ratios. In this study, we describe a novel method for establishing the exact dose and configuration of microsatellite alleles for any accession of an allopolyploid plant species. The method, named Microsatellite Allele Dose and Configuration Establishment (MADCE), can be applied to mapping populations and pedigreed (breeding) germplasm in allopolyploids.

**Results:**

Two case studies are presented to demonstrate the power and robustness of the MADCE method. In the mapping case, five microsatellites were analysed. These microsatellites amplified 35 different alleles based on size. Using MADCE, we uncovered 30 highly informative segregating alleles. A conventional approach would have yielded only 19 fully informative and six partially informative alleles. Of the ten alleles that were present in all progeny (and thereby ignored or considered homozygous when using conventional approaches), six were found to segregate by dosage when analysed with MADCE. Moreover, the full allelic configuration of the mapping parents could be established, including null alleles, homozygous loci, and alleles that were present on multiple homoeologues. In the second case, 21 pedigreed cultivars were analysed using MADCE, resulting in the establishment of the full allelic configuration for all 21 cultivars and a tracing of allele flow over multiple generations.

**Conclusions:**

The procedure described in this study (MADCE) enhances the efficiency and information content of mapping studies in allopolyploids. More importantly, it is the first technique to allow the determination of the full allelic configuration in pedigreed breeding germplasm from allopolyploid plants. This enables pedigree-based marker-trait association studies the use of algorithms developed for diploid crops, and it may increase the effectiveness of LD-based association studies. The MADCE method therefore enables researchers to tackle many of the genotyping problems that arise when performing mapping, pedigree, and association studies in allopolyploids. We discuss the merits of MADCE in comparison to other marker systems in polyploids, including SNPs, and how MADCE could aid in the development of SNP markers in allopolyploids.

## Background

Polyploidy is an integral part of the evolution of all plant species [1]. Several important crop species are polyploids, including bread wheat (*Triticum aestivum*, allohexaploid), cotton (*Gossypium spp*., allotetraploid), potato (*Solanum tuberosum*, autotetraploid) and the very complex sugar cane (*Saccharum spp*., auto-allo-polyploid). The success of polyploids can be ascribed to multiple factors, including their ability to retain beneficial alleles while allowing the generation of novel variation in duplicated alleles and increased vigour through perpetual hybridity [2].

Autopolyploidy is the result of the combination or duplication of multiple genomes that are sufficiently similar to allow for random bivalent pairing and the formation of multivalents during meiosis. This random chromosomal pairing complicates genetic studies, especially mapping studies, and limits mapping studies to mostly simplex alleles that are in the coupling phase. In contrast, allopolyploids are derived either from the merging of differentiated genomes within one zygote [1] or through the gradual diploidisation of an autopolyploid [1,3,4]. These differentiated genomes behave essentially as meiotically independent sub-genomes (homoeologues) that almost exclusively form bivalents between chromosomes of the same sub-genome, resulting in essentially disomic inheritance. Genetic studies in allopolyploids are complex because alleles can be amplified from multiple homoeologues and because some of these alleles are identical (shared) between homoeologues. However, the disomic inheritance expressed by allopolyploids makes them amenable to diploid mapping procedures. In most genetic mapping studies, the pitfall of shared alleles is circumvented by evaluating only alleles for which one or both of the parents are heterozygous [5]. Once initial single parental maps have been created based on 1:1 segregating alleles, the maternal and paternal maps can be integrated using the 3:1 segregating alleles as bridge markers [6]. Although this approach makes genotyping in polyploids reliable and relatively simple, it is highly restrictive and a great deal of information is lost. Furthermore, this approach limits the number of homoeologous loci that can be mapped with individual microsatellite primer pairs when shared alleles are present. The use of microsatellite markers for genetic studies in allopolyploids has several benefits. Because these markers are multi-allelic, they can show as many different alleles as the ploidy level for an individual plant. This allows the simultaneous mapping of several homoeologous chromosomes and the subsequent evaluation of their macro-synteny. With the advent of fluorescent detection techniques for PCR products, it became possible to reliably quantify the abundance of an amplicon in a PCR reaction. The availability of this allele dose information is the first step in the establishment of allelic configurations in polyploids. Several studies have investigated the use of quantitative data to estimate allele dose in autotetraploids [7-12]. The MAC-PR method, which was developed by Esselink et al. [8], uses ratios between the alleles of a single locus within an accession and compares these ratios with those of other accessions in which the same alleles occur together. The presence of different ratios indicates variability in dose for at least one of the two alleles under investigation. Here we propose the MADCE method for dose estimation, which is based on improvements over the MAC-PR method. In MADCE, we also use disomic segregation patterns and virtual reference alleles to refine the dosage estimation. In this paper, we use the MADCE method to determine allelic configurations in the allo-octoploid cultivated strawberry (*Fragaria × ananassa*) and to demonstrate its utility. This crop species has recently been studied extensively in an effort to create linkage maps [13-16]. The results from five microsatellite primer pairs analysed with MADCE are presented to demonstrate its effectiveness for the construction of genetic linkage maps and the determination of the allelic configuration of mapping parents. Extended methodologies are presented for the application of MADCE in pedigreed cultivars and breeding lines. Finally, we demonstrate the value of this method for examining the flow of alleles over multi-generation pedigrees through the Identity-By-Descent concept using the FlexQTL™ [17] and Pedimap [18] software packages.

## Results

### The MADCE procedure for mapping studies

The Microsatellite Allele Dose and Configuration Establishment (MADCE) method is composed of five successive phases. It starts with (I) a qualitative interpretation of microsatellite data, followed by (II) a quantitative assessment of allele doses, (III) an assessment of the initial allele configuration of the mapping parents, (IV) the generation of molecular marker linkage maps, and (V) the final characterisation of the parental haplotypes, including homozygous loci. Below, these steps are elaborated and exemplified through a step-by-step analysis of five microsatellite primer pairs (Table 1) used in a mapping population derived from a cross between the octoploid strawberry cultivars 'Holiday' (H) and 'Korona' (K), for which the original data are given in additional files 1 through 5. Two of these examples are presented in this document, and the other three are provided in additional files 6, 7, 8. It may be useful to keep a printout of Tables 2 and 3 at hand when going through the examples.

**Table 1:** Microsatellite names and sources

Name	Reference	ABI Platform	Study
**ARSFL010**	Lewers et al. 2005	ABI 3730	M, P
**CO817823**	Spigler et al. 2008	ABI 3730	M
**CX661101**	Spigler et al. 2008	ABI 3730	M, P
**PSContig944**	Spigler et al. 2008	ABI 3730	P
**UAFv7500**	Bassil et al. 2006	ABI 3730	M, P
**UFFxa16H07**	Sargent et al. 2006	ABI 3730	M, P

M = used in mapping study, P = used in pedigree study

**Table 2:** Allelic pairs in order of appearance and homoeologue assignment

Microsatellite	Homoeologue Nr	Allele 1 'Holiday'	Allele 2 'Holiday'	Allele 1 'Korona'	Allele 2 'Korona'	Homoeologue after mapping
**ARSFL010**						
	H1	257	234	286	246	A
	H2	269	269	269	269	C
	H3	0	248	0	0	B
	H4	0	0	0	0	D
**Uffxa16H07**						
	H1	298	269	269	306	B
	H2	262	262	262	262	D
	H3	266	266	266	268	A
	H4	279a	279b	273	287	C
**UAFv7500**						
	H1	336	345	330	345	C
	H2	342	348	348	342	A
	H3	342	342	342	333	B
	H4	348	348	348	348	D
**CX661101**						
	H1	212	212	212	212	D
	H2	223	221	223	224	A
	H3	223	220	223	223	B
	H4	204	204	0	0	C
**CO817823**						
	H1	199	203	199	193	B
	H2	216	195	203	195	C
	H3	236	207	209	236	A
	H4	207	207	207	207	D

**Table 3:** MADCE analysis results of mapped microsatellites

Microsatellite	Allele size (bp)	Holiday (H) or Korona (K)	Allele Presence Absence segregation	Actual Ratio Cluster segregation	Mean values of RCs (Ratio Clusters)	Single dose ratio	Holiday Ratio value	Korona Ratio value	Nr of doses present in Holiday	Nr of doses present in Korona	Holiday repulsion allele	Korona repulsion allele	Homoeologue
**ARSFL010**													
	234	H	**1:1**		0, 27	27	26	0	1	0	257	-	A
	246	K	**1:1**		0, 16	16	0	18	0	1	-	286	A
	248	H	**1:1**		0, 4	4	3	0	1	0	0	-	B
	257	H	**1:1**		0, 15	15	12	0	1	0	234	-	A
	269*	H, K	1:0		REF	REF	REF	REF	2	2	-	-	C\D
	286	K	**1:1**		0, 4	4	0	4	0	1	-	246	A
**UFFxa16H07**													
	262	H, K	1:0		REF	REF	REF	REF	2	2	-	-	D
	266	H, K	1:0	**1:1**	17, 39	17	42	14	2	1	-	268	A
	268	K	**1:1**		0, 13	13	0	13	0	1	-	266	A
	269	H, K	**3:1**	**1:2:1**	0, 9, 20	9	10	7	1	1	298	306	B
	273	K	**1:1**		0, 9	9	0	8	0	1	-	287	C
	279	H	1:0	**1:1**	10, 15	10, 15	22	0	2	0	279	-	C
	287	K	**1:1**		0, 6	6	0	5	0	1	-	273	C
	298	H	**1:1**		0, 3	3	3	0	1	0	269	-	B
	306	K	**1:1**		0, 2	2	0	2	0	1	-	269	B
**UAFv7500**													
	330	K	**1:1**		0, 6	6	0	6	0	1	-	345	C
	333	K	**1:1**		0, 3	3	0	3	0	1	-	342	B
	336	H	**1:1**		0, 6	6	6	0	1	0	345	-	C
	342	H, K	1:0	**1:3:3:1**	7,14,19,24	6	16	13	3	2	348	333,348	AB
	345	H, K	**3:1**	**1:2:1**	0, 7, 13	7	7	7	1	1	336	330	C
	348	H, K	1:0	**1:2:1**	13,18,24	6	17	17	3	3	342(A)	342(A)	A D
**CO817823**													
	193	H	**1:1**		0, 22	22	21	0	0	1	-	199	B
	195	H, K	**3:1**	**1:2:1**	0, 16,28	16	17	14	1	1	216	203	C
	199	H, K	**3:1**	**1:2:1**	0, 17,35	17	16	15	1	1	203	193	B
	203	H, K	**3:1**	**1:1:1:1**	0, 9,14,24	9(K1C)14(H1B)	14	9	1	1	199	195	BC
	207	H,K	1:0	**1:1**	8, 13	4	13	8	3	2	236	-	A D
	209	K	**1:1**		0, 8	8	0	8	0	1	-	236	A
	216	H	**1:1**		0, 6	6	6	0	1	0	195	-	C
	236	H,K	**3:1**	**1:2:1**	0, 4, 7	4	4	4	1	1	207	209	A
**CX661101**													
	204*	H	1:0		9	9	15	0	2	0	-	-	C\D
	212*	H,K	1:0		20	10	18	18	2	2	-	-	D\C
	220	H	**1:1**		0, 8	8	7	0	1	0	223	-	B
	221	H	**1:1**		0, 8	8	8	0	1	0	223	-	A
	223	H,K	1:0	**1:3:3:1**	8,16,23,31	7	12	21	2	3	220(B)221(A)	224	AB
	224	K	**1:1**		0, 9	9	0	11	0	1	-	223	A

Repulsion alleles are given if not homozygous

* Homoeologue assignment can be switched. REF = used as reference allele

#### I. Qualitative interpretation of microsatellite data

As a first step, we identify all alleles that segregate in a qualitative fashion, i.e., presence/absence. By filtering for the presence and absence of these alleles, it is possible to identify homologous (repulsion) alleles, thereby forming allelic pairs.

*Example: Microsatellite 1 - UFFxa16H07: *Primer pair UFFxa16H07 amplifies nine different alleles (Tables 2and 3, additional file 2), and six of these segregate qualitatively. Thus, it is possible to establish that five of these six alleles are homologous: allele 273 K is in repulsion to allele 287 K, and 269HK is in repulsion to 298H and 306 K. Qualitatively determined allelic pairs therefore include 273 K-287 K, 269H-298H and 269 K-306 K. No repulsion allele has yet been found for 268 K.

#### II. Quantitative interpretation of microsatellite data

##### a. a.Identification of reference alleles

The ideal reference allele meets the following criteria: No variation in dose, present in all progeny and not influenced by stutters from other alleles. Stable references for all progeny come from alleles that are homozygously present in one or both parents (i.e., AA × aa or AA × AA, not AA × Aa). Usually, these alleles can only be distinguished through the use of other initial, less optimal reference alleles, such as simplex alleles that segregate 1:1 for presence and absence.

*Example: Microsatellite 1 - UFFxa16H07: *Allele 262HK is present in all progeny and in both parents. Ratio clusters with several 1:1 presence/absence-segregating alleles reveals that allele 262HK does not segregate by dose and is therefore homozygous in both parents. This allele can thus be used as a reference allele.

##### b. Ratio calculation, cluster identification

Allele doses are estimated by examining the peak area of an allele relative to the peak area of the reference allele. The resulting Ratio Values (RVs) are plotted in frequency distributions (see additional files 1, 2, 3, 4, 5). When the reference allele performs well, the RVs show an apparently normal frequency distribution around a certain mean RV. This cluster of ratio values is called a ratio-cluster (RC). The identification of more than one clear RC in the progeny implies the presence of dosage variation, which in turn indicates segregation. Narrow, non-overlapping frequency distributions around RCs indicate that the reference allele performs well. The obtained RVs for each allele are multiplied by an empirically obtained factor to make the range of RVs similar for each allele and facilitate the generation of frequency distributions.

**Figure 1:**
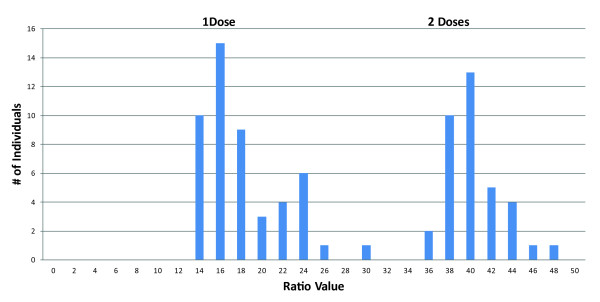
**Example of the use of Ratio Value frequency distributions**. Frequency distribution of ratio values for allele 266 of microsatellite UFFxa16H07 in the progeny from octoploid strawberry cross H × K.

*Example: Microsatellite 1 - UFFxa16H07: *Alleles 266HK and 279H are always present in all progeny. 266HK has two RCs with mean values of 17 and 39 (Table 3) and cluster distributions that allow dosage quantification (Figure 1), reinforcing the suitability of 262HK as reference. The RCs show regular steps, with the higher mean approximately two times greater than the lower mean. 279H also shows two RCs with mean values of ten and 15 (Table 3) and thus also segregates in dose.

##### c. Segregation of Ratio Clusters

Segregation patterns of ratio clusters (RCs) are established by comparing the number of individuals in a certain RC to the numbers in other RCs. Common RC segregation ratios for presence/absence alleles are: 1:1 (Aa × aa), 1:2:1 (Aa × Aa) and 1:3:3:1 (AaBb × Aabb, where A and B are homoeologues). These ratios also occur for alleles that are always present due to homozygosity in at least one of the parents but that still segregate in terms of dosage. For instance, the RC segregation ratio of 1:2:1 for an allele that is always present (and therefore must be present on more than one homoeologue) can be caused by several parental allelic configurations, such as AaBB × aaBb, AAbb × AaBb, AABb × aaBb and AABb × AABb. The allele doses of these parents are 3 × 1, 2 × 2, 3 × 1 and 3 × 3, respectively.

*Example: Microsatellite 1 - UFFxa16H07: *The two clusters of both 266HK (Figure 1) and 279H segregate in a 1:1 pattern (Table 3).

##### d. Identification of homologous alleles

Homologous allele pairs are identified in a manner similar to the qualitative method, but allele dose is considered instead of presence and absence. If an allele is present in its highest dose, this will automatically lead to a decrease in dose or absence of the homologous allele, and vice versa. If no such allele is found, it is likely that the repulsion allele is a null allele.

*Example: Microsatellite 1 - UFFxa16H07: *The highest dose of 266HK leads to the absence of simplex allele 268 K, for which no repulsion allele could be found through qualitative analysis. This led us to establish 266 K-268 K as an allelic pair. Because 266HK is present in all progeny, our analysis on the two previous homologous 'Korona' alleles implies that 'Holiday' is 266-homozygous. We have now completed the allelic pairs for all four 'Korona' homoeologues and three out of the four 'Holiday' homoeologues. The only remaining allele to be analysed is 279H, which will be addressed in the next section.

When one or both parents have multiple doses of an allele, it is necessary to establish the dose of each allele to properly genotype individuals and identify allelic pairs. Allele doses are estimated by examining the peak area of an allele relative to the peak area of other allele(s) from the same PCR amplification, similar to the procedure published by Esselink et al. [8]. A large, consistent variation in the ratio between the peak areas of two different alleles indicates the segregation of allele dose for one or both alleles. The quantitative interpretation method is divided into four stages, which are described in the following sections.

#### III. Determining parental allele configuration and cross checking

The purpose of this step is to determine the allelic configuration of the mapping parents through their progeny. These configurations are subsequently validated using the estimated allelic doses of the parents and vice versa. First, maternal allele pairs are matched with their paternal homologues using the alleles shared between parents. For example, if one parent (P1) has a homozygous allele of size (223,223) and the other parent (P2) has a heterozygous pairing between 223 and 225, (223,225), then the allele pairs P1(223,223) and P2(223,225) are considered homologous. Similarly, if an allele is homozygous in both parents (e.g., P1(229,229), P2(229,229)), these pairs are also considered homologous. When no alleles are shared between parents, matching can sometimes be achieved based on other criteria, such as differences in amplification efficiency between homoeologues or differences in the range of allele sizes. Additionally, when three sets of matching parental allele pairs have been identified for an allo-octoploid, the fourth set automatically consists of the last two remaining allele pairs. The process of matching parental allele pairs is continued until all sets are matched; these sets represent the homoeologues. Next, the number of obtained allelic sets is compared with the number of allele sets expected based on the ploidy level. If fewer sets are obtained, one of the homozygous alleles may actually be present in two homoeologues (A & B), e.g., P1(229A,229A)P2(229A,229A) + P1(229B,229B)P2(229B,229B). Alternatively, there may be a homoeologue that contains only null alleles, such as P1(229A,229A)P2(229A,229A) and P1(0B, 0B)P2(0B, 0B). Establishing whether a homoeologue is homozygous null or homozygous for a shared allele can be difficult. Examining the consistency of the allele doses inferred between the parents and progeny may be helpful, as long as the alleles have similar amplification efficiencies. For example, in an AAxAa cross, the RV of the mother should be approximately twice that of the father (2:1), and these RVs should be in agreement with the two RCs of the progeny (AA, Aa). If the RVs have a 4:3 relative value, this could indicate the presence of an additional homozygous set of allele pairs that had not been initially identified (i.e., AABBxAaBB). In some cases, the number of alleles can exceed the ploidy level. In such situations, indications for duplications or amplification efficiency differences should be examined.

*Example: Microsatellite 1 - UFFxa16H07:*Based on shared alleles (262HK, 266HK, 269HK) we can group the allele pairs of 'Holiday' and Korona into three sets of homoeologues (Table 3). The fourth set of homoeologues depends on the analysis of 279H. This allele shows two RCs and thus segregates by dose. However, the presence of a high dose of 279 is not associated with the absence of any other allele. Therefore, a logical hypothesis is that the homologous allele for 279 is a null allele and that 279 is homozygous on another homoeologue. However, this conclusion is problematic because it results in nine allele doses in a single parent ('Holiday')-six allele doses from the three allele pairs and three doses from 279-but only eight doses are possible in an allo-octoploid (when alleles are non-duplicated). An alternative is to assume the presence of two homologous alleles that differ in amplification efficiency (279a and 279b). The decrease in amplification efficiency leads to a low mean RC value (10) for one allele and a high mean RC value (15) for the other allele (Table 3). Because they are homologous, one of them is present in all progeny. Further evidence corroborating this hypothesis is that the RV of 279 in the 'Holiday' cultivar is much higher than the highest RC mean of the progeny; if the first hypothesis is correct, the 'Holiday' RV value should be similar to the highest RC mean of the progeny. In the alternative interpretation, the total dose in both 'Holiday' and 'Korona' produces eight doses per parent. Therefore, the 279 allele pair 279aH-279bH is joined with the last remaining 'Korona' allele pair, 273 K-287 K.

### A virtual reference allele: Increasing throughput and power

#### Principle

The reference allele in the example above is based on a single allele. Using single allele based references can be a laborious procedure, especially if no monomorphic, homozygous loci that are not confounded by stutter bands can be found. Throughput can be increased considerably for most microsatellites through the use of a virtual reference allele that is based on the average of all, or part of, the allele peak areas for a primer pair in an individual. Automated calculation of averages and Ratio Values enables rapid dosage assessments. Because it is based on multiple alleles, a virtual reference allele diminishes the risks of inaccurate area estimation for individual alleles and reduces the impact of stutters influencing single peaks. Paradoxically, this makes dosage estimation better with increasing ploidy levels because the average is based on a larger number of observations.

#### Checking performance

Checking the performance of the virtual reference is best achieved by checking the narrowness of the RC distributions generated for alleles of known dose. If their width is narrow, the designed virtual reference is adequate. If they are wide, the interpretation of the data could be hampered. In that case, further optimisation of the virtual reference can usually be performed by accounting for putative interfering factors, such as segregating null alleles and large amplification differences between alleles. Interfering alleles can be identified by excluding suspect alleles from the average and then checking for improvement in the narrowness of the RC distributions.

#### Impact of differences in allele amplification efficiency

Efficiency of PCR amplification decreases with increasing allele size [10]. Furthermore, the presence of mutations in the primer sites can have a large influence on amplification efficiency. Consequently, homologous simplex alleles may have a many-fold difference in peak area (here, two- to three-fold, as shown in Table 3). This can have a significant influence on the reliability of dose estimation. As an illustration, assume a primer pair amplifies four different alleles over a tetraploid mapping population. Seven of the eight parental alleles amplify with equal efficiency, whereas the eighth allele has a three-fold higher efficiency. If this difference in amplification is not accounted for, the average of half of the tetraploid progeny represents six 'amplification units' ((3 × 1) + (1 × 3)), and the other half represents four units (4 × 1). The average-based reference in individuals carrying the efficient allele will thus be 50% higher than in individuals lacking this allele. This variability in the reference will greatly affect the width of the ratio clusters and may lead to false interpretations. In cases of too-wide RCs, using a subset of alleles with similar amplification efficiencies usually improves the width of the reference allele considerably. If this approach still does not suffice and if the involved markers are of great interest, a more sophisticated but also much more laborious approach may be considered: the introduction of allele-specific multipliers. Such multipliers are calculated for strongly deviating alleles based on their ratio to an initial reference allele with a known dosage. The inverse of the mean value of this ratio across the progeny can then be used as multiplier in the calculation of the virtual reference.

**Figure 2:**
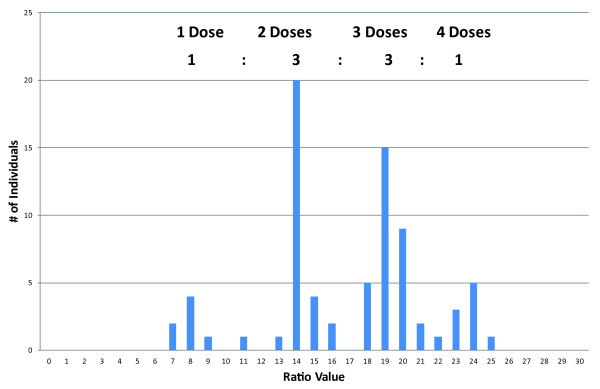
**Example of the use of the Ratio Value frequency distributions**. Frequency distribution of ratio values for allele 342 of microsatellite UAFv7500 in the progeny of octoploid strawberry cross H × K. The relative amplification ratio was calculated against a virtual average-based reference allele.

*Example: Microsatellite 2 - UAFv7500*: Primer pair UAFv7500 amplifies six different alleles, four of which segregate by presence/absence (Tables 2 and 3, additional file 3). Qualitative analysis reveals three 1:1 segregating alleles (330 K, 333 K, 336H) and one 3:1 segregating allele (345HK). 345HK is allelic with both 330 K and 336H, indicating that they belong to the same homologous set (336H-345H, 330 K-345 K). No allelic pair can be found for 333 K. Using the simplex alleles as references, all of the alleles that are always present segregate by dose. This makes it impossible to use a single peak as a reference for all the samples. Therefore, an average-based virtual reference allele must be constructed. Examination of the RCs of the simplex alleles showed narrow distributions (additional file 3), and thus, no further optimisation of the reference allele is required. Next, 345HK shows a 1:2:1 RC segregation, which confirms the qualitative analysis of 3:1 segregation. 342HK and 348HK are always present, indicating the presence of at least one homozygous locus for each. 342HK shows four RCs of regular increases (approximately 2×, 3× and 4× the ratio of 6) and a 1:3:3:1 segregation (Table 3, Figure 2). In allopolyploids, this segregation pattern indicates triple heterozygosity, for which at least three heterozygous allele pairs must be involved in at least two homoeologues. Allele 348HK segregates 1:2:1 in RCs, indicating double heterozygosity. When samples are filtered for the highest RC of 342HK, the single-dose 333 K allele is absent, and 348HK is at its lowest RC value. In contrast, when samples are filtered for the lowest RC of 342HK, 333 K is always present and 348HK has its highest RC value. 342HK is thus fully complementary to 333 K (333 K-342 K), and both of the other two segregating 342HK alleles are complementary to both of the segregating 348HK alleles. Next, the homozygous alleles must be assigned to their parents. To accomplish this, the ratio values of the parents are explored. For 342HK, these ratios indicate the presence of three doses in 'Holiday' and two in 'Korona' (Table 3). Because one of the two 342 alleles of 'Korona' is known to segregate due to its complementarity to 333 K, the second 342 K allele also has to segregate. The homozygous 342 locus and the third segregating 342 allele must thus originate from the 'Holiday' parent. The deduced allelic composition of the two involved homoeologues is thus (342H-342H, 333 K-342 K), (342H-348H, 342 K-348 K). This completes the analysis for 342HK.

At this point, the homozygous 348 allele has not yet been assigned and is assumed to be present on the 4^th ^homoeologue. Because no other unassigned alleles are available, both parents may be 348-homozygous, or one may be homozygous null. The parental ratio values are helpful to distinguish these two options. They indicate the presence of three doses in each parent (Table 3), whereas only one segregating allele per parent has been assigned based on the segregation patterns of the progenies. This indicates that both parents are homozygous for 348 at the fourth homoeologue.

#### IV. Mapping and Validation

Having assigned alleles to homologous sets, we can now begin mapping. The mapping step serves five purposes: to validate and complete the assignment of allelic pairs, to group allelic pairs from multiple loci together into different homoeologous sub-genomes, to determine marker order and genetic distances, to establish an integrated map, and to determine the parental haplotypes. The mapping step is divided into four stages that are similar to the map integration procedure described by Barrett et al. [19]. The final validation of the MADCE-derived allele scores and homoeologue assignments is accomplished through the generation of linkage maps.

##### a. Creation of a priori integrated loci

The single parental allelic sets identified during the previous allele configuration step (step III) are combined into bi-parental sets and then translated into integrated loci. In our case, these loci were defined as Cross Pollinator (CP)-type loci in the software JoinMap (Kyazma B.V., Wageningen, NL). The integrated loci are constructed prior to mapping for two reasons. First, this serves as an additional check for scoring errors (e.g., individuals with three alleles for one locus). Second, it facilitates data-management; it is much more efficient to generate integrated loci first and use JoinMap 4.1 to convert them back to single parent loci when needed than to integrate loci at a later stage.

##### b. Creation of separate parental maps: additional error checks

To create separate parental maps, integrated loci are automatically converted into single-parent loci in JoinMap 4.1. After the maps are generated, a number of standard error checks are performed, such as comparison to a reference map (for strawberry FvxFb [20]) and a check for distorted loci and loci that create high tension. Finally, marker genotypes are conditionally formatted in Excel using the phase information from JoinMap, which enables the easy identification of discover double crossover events.

##### c. Creation of separate parental maps: validation of integration and homoeologue assignment

Next, a new grouping is calculated and maps are drawn. The single parental maps are matched to each other based on allele sharing and can be used to validate integrated loci and create new ones from previously unintegrated loci, as described by Barrett et al. [19]. The pairs of matching parental maps are then randomly assigned a homoeologue letter (A, B, C, or D in the case of an octoploid).

##### d. Creation of integrated maps: final error check

The upgraded data from the previous step are loaded into JoinMap, and the final integrated maps are generated. The map and linkage phase information generated by JoinMap is combined with the allelic information from step III to establish the parental haplotypes.

**Figure 3:**
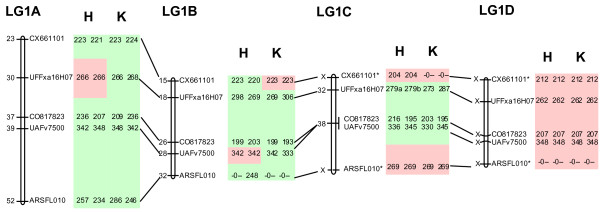
**Linkage maps for the four homoeologous chromosome pairs of LG1 in the allo-octoploid strawberry**. These maps are based on the five microsatellites that have been used to exemplify the MADCE approach for the assessment of allele doses and assignment of sub-genomes based on parent-specific allelic pairs. Positions (in cM) are shown on the left side of each linkage group (LG). Positions with an × instead of a value are homozygous in both parents and therefore cannot be used for determining map distances. A green background indicates heterozygosity, and a red background indicates homozygosity. H and K indicate the 'Holiday' and 'Korona' parents, respectively. * Allelic configurations are interchangeable between the related homoeologues. 0: inferred null allele.

*Example: *The final marker scores of the five example primer pairs (Table 1) for use in JoinMap are given in Additional files 1, 2, 3, 4, 5. The mapping results and final haplotypes are presented in Figure 3. The marker order is the same for all homoeologues, although some contain fewer or no segregating loci. Graphical analysis of the marker data shows the absence of any double recombination event over short distances, indicating that the quantitative interpretation of the microsatellite data was successful, as it led to unambiguous data. MADCE thus resulted in highly robust marker genotypes.

#### V. Completion of parental haplotypes for homozygous loci

If the presence of homozygous loci becomes evident in step III, the loci are manually added to the haplotype information to make the final haplotypes complete. Their genetic position is extrapolated or interpolated from the distances between markers on homoeologous genomes. These positions should not be considered biologically "true", but they can serve as a guideline to allow visualisation of homozygous stretches.

*Example: *In the current study, one of the homoeologues (1D) is shown to be completely homozygous (Figure 3).

### MADCE procedure for pedigreed cultivars

**Figure 4:**
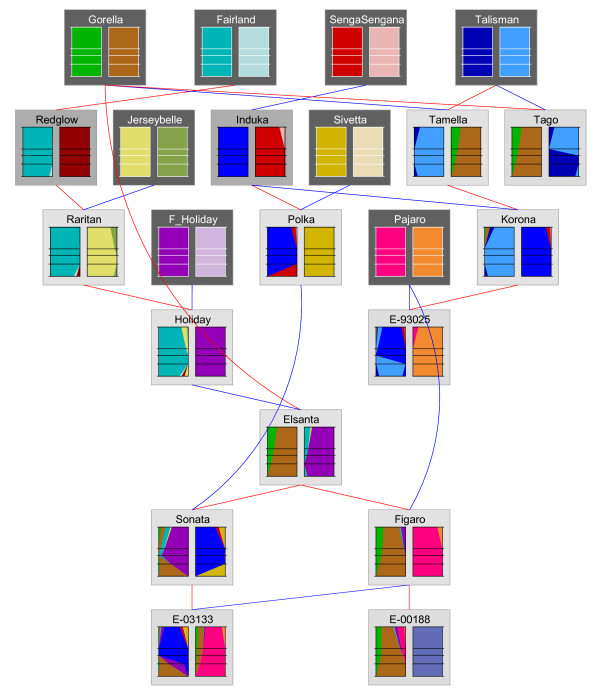
**Genetic relationships among the 21 pedigreed strawberry cultivars and breeding lines, and a graphical representation of FlexQTL IBD (identity-by-descent) probabilities for LG1C**. Dark grey, medium grey and light grey boxes represent cultivars for which none, one or both parents are included in the current survey. Haplotypes are represented by the coloured boxes, and each colour represents a different founder haplotype. Red lines indicate maternal parents and blue lines indicate paternal parents. Horizontal lines within a coloured box represent the positions of five different marker loci (from top to bottom: PSContig944, CX661101, UFFxa16H07, UAFv7500 and ARSFL010). The width of a colour at a particular height reflects the probability that the corresponding founder allele is present at that locus on the map (see Voorrips 2007).

The MADCE procedure for mapping studies is not directly applicable to pedigreed cultivars because of their higher genetic complexity (e.g., a greater number of alleles per locus) and the very limited availability of segregation data (few progeny per individual). Here, we will describe the MADCE procedure as adapted for pedigreed cultivars and illustrate this procedure for microsatellite CX661101 (Table 4) using a set of 21 pedigreed cultivars (Figure 4).

**Table 4:** Dose assessment, Ratio Values and homoeologue assignment for CX661101 in a pedigreed set of cultivars

Allele		204	210	212	218	220	221	223	224	225	229	Dose Sum	Null Alleles
**Homoeologue Assignment**		**C**	**C**	**D**	**D**	**B**	**A**	**AB**	**A**	**B**	**A**		**C**

**E-00188**	D	**1**	**1**	**2**			**1**	**1**		**2**		**8**	
	RV	6	6	14			6	5		6			
**E-03133**	D	**1**		**2**				**3**		**1**		**7**	**1**
	RV	6		14				20		4			
**E-93025**	D		**1**	**2**				**3**	**1**			**7**	**1**
	RV		4	17				18	5				
**'Elsanta'**	D	**2**		**2**				**2**	**1**	**1**		**8**	
	RV	11		15				10	4	3			
**'Fairland'**	D	**2**		**1**	**1**			**3**	**1**			**8**	
	RV	9		7	6			16	5				
**'Figaro'**	D	**2**		**2**				**3**		**1**		**8**	
	RV	10		15				19					
**'Gorella'**	D	**2**		**2**				**2**	**1**	**1**		**8**	
	RV	9		14				12	5	3			
**'Holiday'**	D	**2**		**2**		**1**	**1**	**2**				**8**	
	RV	9		13		6	6	11					
**'Induka'**	D	**1**		**2**				**3**	**1**			**7**	**1**
	RV	6		16				15	7				
**'Jerseybelle'**	D	**2**		**2**			**1**	**3**				**8**	
	RV	9		14			6	15					
**'Korona'**	D			**2**				**3**	**1**			**6**	**2**
	RV			18				18	8				
**'Pajaro'**	D	**1**	**1**	**2**				**2**	**1**	**1**		**8**	
	RV	5	5	15				11	5	3			
**'Polka'**	D	**1**		**1**	**1**			**2**	**2**			**7**	**1**
	RV	7		7	7			12	11				
**'Raritan'**	D	**2**		**2**			**1**	**3**				**8**	
	RV	10		13			6	15					
**'Redglow'**	D	**1**		**2**				**3**	**1**			**7**	**1**
	RV	7		16				16	5				
**'Senga S.'**	D		**1**	**2**				**3**	**1**			**7**	**1**
	RV		5	15				17	6				
**'Sivetta'**	D	**2**		**1**	**1**			**1**	**1**	**1**	**1**	**8**	
	RV	11		8	6		1	4	5	3	6		
**'Sonata'**	D	**1**		**1**	**1**			**3**	**1**			**7**	**1**
	RV	6		8	7			18	5				
**'Tago'**	D	**1**		**2**				**3**			**1**	**7**	**1**
	RV	5		16				17			6		
**'Talisman'**	D	**1**		**2**				**3**			**1**	**7**	**1**
	RV	5		14				19			6		
**'Tamella'**	D	**1**		**2**				**4**				**7**	**1**
	RV	6		16				23					

*D *dose, *RV *ratio value. Homoeologue assignment of the underlined homoeologue letters was performed based on results from the HxK mapping population

#### I. Allele dose estimation requires a virtual reference

We recommended using virtual, average-based reference alleles because single alleles that fit the requirements for a reference are very rare in a large breeding germplasm. Ratio value (RV) calculation, frequency distribution and dose assignment are the same as for mapping populations. When an RV falls between two different clusters, it can be provisionally assigned the most likely dose and verified during step II. The occurrence of null alleles is more likely over a wide germplasm. Null allele doses are determined by subtracting the total dose estimated from the total dose expected (i.e., the same as the ploidy level). A separate column for null allele doses is added to the spreadsheet (Table 4). These null alleles can be assigned to homoeologues in a fashion similar to regular allele assignment, through the method described in the next section. For pedigreed cultivars, null alleles cannot be compensated for a priori. Allele efficiency differences can only be compensated for with multipliers when they are very clearly present and can be quantified. These drawbacks make dosage estimation somewhat less reliable in cases where these pitfalls are present, but it is not impossible.

*Example: *For microsatellite CX661101, an average-based reference is created. The presence of distinct RV-clusters indicates the occurrence of different dose levels and a good performance of the virtual reference. The regular distances between RV clusters indicate the absence of very large differences in amplification efficiency between alleles (Table 4). For allele 204, we observe one to two doses with RCs, near RVs 5 and 10, respectively. Allele 212 is always present and also shows two different RCs (~8 and ~16), again suggesting a single vs. a double dose. Allele 223 has RVs ranging from 4 to 23, suggesting a more complicated situation. The RCs appear to increase and decrease in steps of approximately 5-6 (although the number of observations per cluster is quite low). This step size is therefore likely to represent the single dose value. From this we infer the dose levels of each cultivar, which ranges from 1-4 doses. Because we have mapped this microsatellite in 'Holiday' and 'Korona' (Table 3), we can use these cultivars as a check for correct dose estimation, and we find that the results are in agreement. Now, we can proceed to infer null allele doses, which we know to be present from the mapping study. The total dose of amplified alleles ranges from six to eight (Table 4), therefore, the total dose of null alleles ranges from two ('Korona') to zero. Not surprisingly, the ratio values for 212 K and 224 K are the highest of those in all cultivars and selections (after taking into account allele dose). This is due to the presence of the two null alleles, which led to a relatively low virtual reference value and therefore high ratio values.

#### II. Assigning alleles to homoeologues

##### a. Procedure: Assignment by total homoeologue allele dose

A very simple rule for disomically inheriting species is that the total dose of all alleles belonging to the same homoeologue in any given individual needs to be exactly two (null allele doses are also used in this calculation). If an allele of the same size is shared by multiple homoeologues, the total dose needs to be two times the number of homoeologues involved. These rules are a good starting point for situations in which no prior allele assignment from previous mapping studies is available. They are especially useful for large data sets and for alleles that occur at frequencies of > 5%. We begin the allele assignment process by choosing the first allele and then eliminating alleles that cannot be on the same homoeologue because the sum of their combined doses exceeds two. It is easiest to start with a high-frequency allele that varies in dose from zero to two. Alleles for which the summed dose never exceeds two are likely to be allelic to each other. This analysis is repeated starting with another unassigned allele to determine the composition of the other homoeologous series.

*Example: *We assume that there is no prior allele assignment information from mapping populations, except for the naming of the homoeologues. We begin the analysis with allele 204. This allele segregates by presence/absence and, when present, occurs at either a single or double dose (Table 4). Assuming that 204 occurs on a single homoeologue, we can, for each genotype, sum its dose with those of each of the other assumed homoeologue-specific alleles. Alleles that lead to a sum that is higher than two in any individual do not belong to the same homoeologue. This is the case for all alleles except 210 and the null allele. Summing the doses of these three alleles results in all genotypes having exactly two doses, indicating that allele assignment for this homoeologue is complete. This set of alleles is assigned homoeologue letter C based on the results of the mapping study.

We continue with a similar analysis of allele 212. The total dose exceeds two in combination with all alleles, except for 218. This allele is only present when 212 is at single dose, so 212 and 218 are likely to be homologous. The total dose sum for 212 and 218 is exactly two for all cultivars, and therefore, assignment for this homoeologue is complete. This set of alleles is assigned homoeologue letter D based on the results of the mapping study.

We observe that allele 223 is always present and occurs at up to four doses. It must therefore be present on at least two homoeologues. Because two homoeologues have been assigned already, 223 must be present on the other two (A&B). The remaining alleles must therefore be present on these two homoeologues as well. Of these, only 225 and 224 occur at a double dose. The assumption that double-dose (maximum) alleles are present on only one homoeologue eliminates the possibility that 221 is on the same homoeologue as 225 because they add up to three doses in selection E-00188. Allele 221 is present on homoeologue A in 'Holiday', so we assign 225 to homoeologue B. For double dose allele 224 ('Polka'), no eliminations can be achieved because 'Polka' carries only the shared allele 223. The remaining alleles (220, 224, 229) cannot be assigned through the dose procedure.

##### b. Procedure: Assignment by transmission

To help in completing or validating assignments, we can consider allele transmission from parents to progeny. For alleles that are on the same homoeologue, only one allele should be transmitted. Simultaneous (non)transmission of alleles indicates that they belong to different homoeologues. By tracking the transmission of alleles over a large pedigree, it is possible to establish which alleles definitely do not belong to the same homoeologue and which alleles are likely to belong to the same homoeologue. The above principle does not apply to alleles that are shared between homoeologues.

*Example: *Using the pedigree shown in Figure 4 in combination with the data in Table 4, we observe that alleles 224 and 225 are both present in 'Gorella' and that neither of these alleles are transmitted from 'Gorella' to 'Tamella'. This indicates that these two alleles are present on different homoeologues. This is corroborated by the transmission from 'Gorella' to 'Elsanta', in which both are transmitted and neither could have come from 'Holiday', which is the other parent of 'Elsanta'. Because 225 was assigned to homoeologue B, we can now assign 224 to homoeologue A. Similarly, we find that 221 and 220 are both present in 'Holiday', but neither are transmitted to 'Elsanta'. This means that 220 is not homologous to 221 and therefore must be on homoeologue B. This leaves only 229 unassigned; this allele is present in 'Sivetta' along with 225 of homoeologue B. Because neither of these two alleles is transmitted to 'Polka', 229 must be present on homoeologue A. We thus obtained two allelic sets: 221-223-224-229 for homoeologue A and 220-223-225 for homoeologue B.

Once all alleles have been assigned, we can genotype the A and B homoeologues for the 223 allele. For instance, 'Polka' has 223 and 224 at a double dose. Because 224 occurs only on the A genome, 'Polka' must be homozygous for 224 on the A homoeologue and must thus be homozygous for 223 on the B homoeologue. 'Elsanta' has 223 at double dose and 224 and 225 at single doses. Alleles 224 and 225 belong to different homoeologues. Therefore, the double dose of 223 must come from the two different homoeologues (223A-224A, 223B-225B). Finally, we confirm whether the assignments are consistent with those of 'Holiday' and 'Korona' from the mapping population.

Results for four other example microsatellites:A similar analysis has been performed with five other microsatellites (Table 1). For each, a summary of the resulting allelic sets is presented in Table 5. For one microsatellite (CO817823), we encountered difficulties in dose estimation in a few cultivars, and it was not possible to complete the analysis of this microsatellite. Twelve alleles occurred at multiple doses (seven at 2×, four at 3×, and one at 4×), all of which could be assigned to specific homoeologues.

**Table 5:** Microsatellite alleles observed in 21 cultivars of the allo-octoploid strawberry for six microsatellite markers and their assigned to the four homoeologues

Microsatellite	LG1A Diversity	LG1B Diversity	LG1C Diversity	LG1D Diversity	Max dose observed
**ARSFL010**	234,242,246,257,286	null* (248 in 'Holiday')	244,**248**,252,259,264,266,269	null	2× (234,257,269)
**CO817823**	205,207?,209,211?, 236	null?,193,199,**203**, 209?,211?	null?,195,**203**,216	null,205?,207, 210?	unknown
**CX661101**	221,**223**,224,229	220,**223**,225	null,204,210	212,218	4× (**223**)
**PsContig944**	null,**115**,152,156,**160**, 161,162,169,173	**115?**,155,**160**,166,168, 179,181,184	**115**,137	152,158	3× (**115**)
**UAFv7500**	**342,348,351**	333,**342,345**	330,336,**345**	339,**345,348,351**	3× (**342,345,348**)
**UFFxa16H07**	266,268	269,275,286,298,306	271,273,279,287	262	2× (262,266,273,279)

Alleles in bold occur at multiple homoeologues. Alleles followed by a question mark have uncertain assignment.

* Allele 248(HomB) was observed during mapping, but due to its low amplification it was not detected in the set of pedigreed cultivars.

With dose estimation completed, we can proceed to identify allelic pairs that belong to the same homoeologue (homologous alleles). This is best achieved by combining two methods, one based on allele dose and one on allele transmission.

#### III. Phase determination and assignment validation using FlexQTL™

The data are now used to generate linkage phase and Identity-By-Descent (IBD) estimations using the FlexQTL™ software [17]. FlexQTL™ also monitors the number of observed and expected single and double recombination events between successive markers, making this software an easy tool with which to quickly validate assignments and check for alternatives (erroneously assigned alleles lead to an increased number of apparent recombination events throughout the pedigree). Evidently, proper phase estimations can only be made in cases where a founder has sufficient offspring.

**Figure 5:**
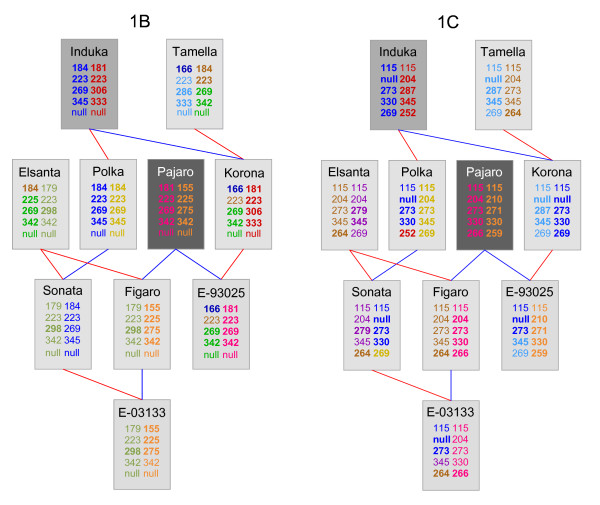
**Subsection of the pedigree from Figure 3 showing the sizes and the most probable parental origins of alleles for the loci of LG1B (left) and LG1C (right)**. Red lines indicate maternal and blue lines paternal parents. Each horizontal pane describing allele sizes represents a different microsatellites (from top to bottom: PSContig944, CX661101, UFFxa16H07, UAFv7500 and ARSFL010). Different colours represent different founder haplotypes. Alleles in bold have an IBD value of > 0.9. Dark grey, medium grey and light grey boxes represent cultivars for which none, one or both parents are included in the current survey.

*Example: *Figure 4 presents the haplotypes obtained for the 21 studied cultivars. Figure 5 demonstrates the flow of individual alleles over the pedigree for a subset of these cultivars. The pedigree-derived haplotypes for 'Korona' are consistent with those determined by the mapping population (Figure 3 and 5). This is also the case for the 'Holiday' cultivar (data not shown), thus delivering proof of concept of the suitability of MADCE for the genotyping of pedigreed germplasm in allopolyploid crops.

## Discussion

### Methodological aspects of MADCE

A variety of allele dose estimation techniques have been examined in the past decade. The methods employed include visual evaluation of intensities [9,21,22], peak height [10] and peak area ratios (MAC-PR) [8,11]. MAC-PR [8] is likely to be the most accurate of these due to its use of an internal reference that allows compensation for PCR efficiency variation between samples. MADCE improves upon this method with the new concept of virtual (average-based) reference alleles and extends it with a pipeline for data analysis for the assessment of the full allelic configuration of allopolyploid genotypes. The robustness of MADCE is assured thanks to the presence of several internal feedback controls that monitor the accuracy and consistency of dosage information.

#### Virtual reference allele

The throughput and reliability of dose estimation is heavily dependent on the kind and quality of the reference allele and the "overall" quality of the microsatellite (stutters, peak shape, allele size proximity). The use of "virtual" average peak-area-based references is recommended for both mapping studies and pedigreed sets of cultivars because they are robust, are widely applicable and increase throughput. Virtual references are more robust because they are less sensitive to the quality of individual peaks and stutters. Virtual references increase throughput because a single fully informative reference can be used instead of a series of partially informative references, which would require more data handling. The performance of the virtual reference can be easily monitored by measuring the width of RC clusters, and the need for additional, performance improving measures can easily be detected. MADCE's use of a virtual reference provides a basis for semi-automated analyses including the development of dedicated software for dose estimation [12], thus enabling further increases in throughput in the future.

#### Homoeologous amplification

Within allopolyploids, primer pairs can potentially amplify products from one or all of the homoeologues and thus may yield a large number of alleles with complex banding and segregation patterns. This could be seen as a disadvantage because it hampers quick and easy data interpretation, especially in the assignment of alleles to specific homoeologues. Alternatively, it could be seen as an advantage because it allows the examination of macro-synteny. In addition, fewer primer combinations are needed relative to methods that use homoeologue-specific primers.

The main disadvantage of homoeologous amplification is that alleles that are exactly the same size between multiple sub-genomes can occur. These shared alleles cause most of the complex segregation patterns in allopolyploids. Shared alleles could indicate the conservation of microsatellite repeat number between homoeologues but could also have arisen independently through different events (homoplasy) [23-25]. The case for homoplasy is corroborated by the fact that in our study, one of these shared alleles shows a difference in amplification efficiency (Table 3), indicating the presence of polymorphisms such as indels or SNPs at the primer annealing site, these types of polymorphism have lower mutation rates than repeats. Using MADCE, it is possible to tackle these obstacles by dissecting all alleles into simple Mendelian segregation patterns and assigning the alleles to their respective sub-genomes, despite their size similarities.

#### Improvement of mapping efficiency by MADCE

In our case study of the octoploid strawberry, five microsatellite primer combinations amplified 35 different alleles based on size, and 25 of these exhibited presence/absence segregation. Using MADCE, we uncovered 30 highly informative segregating alleles, whereas a conventional approach would have yielded only 19 fully informative and six partially informative alleles. Of the ten alleles that were present in all of the progeny and thereby ignored or considered homozygous when using conventional approaches, six were found to segregate by dosage. Additionally, the information content of the six 3:1 segregating markers increased because MADCE allows the discernment of homozygous and heterozygous progeny, thus doubling the proportion of informative meioses from 25% to 50%. By identifying the matching repulsion alleles, all four genotypic classes could be distinguished, thus increasing the meiotic information content to 100%. Moreover, one 3:1 segregating allele of one of these microsatellites could be correctly assigned to different homoeologues. If this allele had been used as a bridge marker for parental map integration, according to the protocol defined by Ripol et al. [6], this would have led to a false integration that would have linked homoeologous instead of homologous chromosomes.

#### Improvement of mapping quality by MADCE

The identification of homologous (repulsion) alleles and the creation of a single locus for both alleles prior to mapping have been used previously [14]. This reduces the number of redundant loci, some of which might have looked like different loci at slightly different map positions due to inconsistencies in scoring or missing scores. The error removal steps of MADCE improve the final map quality. Falsely integrated parental maps that occur due to shared alleles are prevented by adequate allele assignment to the different homoeologues, as with the map integration method described in Barrett et al. [19]. The determination of the full allelic configuration of mapping parents by MADCE allows the easy identification of regions in the genome that are completely homozygous, as demonstrated for linkage group 1D. Information about the presence of large homozygous segments can reveal gaps and the occurrence of an excessive number of partial linkage groups when making linkage maps in outbreeding plant species. This information can thus prevent futile efforts to fill these gaps or complete these linkage groups by testing numerous additional primer combinations.

### Enabling pedigree-based analysis

In allopolyploids, association studies are still in their infancy, despite the fact that many economically important crops, such as wheat and cotton, are allopolyploid. And despite that some of these plants have well-diverged sub-genomes [26-28] and propagate through inbreeding, which make them genetically less complicated. Over the last decade, there has been a shift from bi-parental QTL mapping studies towards studies on preferably unstructured plant germplasm through LD mapping [29]. Additionally, strategies have been developed for genetically structured breeding germplasm through a pedigree-based analysis (PBA) approach [17,30]. Both approaches offer perspectives for allopolyploids.

PBA is a QTL mapping approach for multiple pedigreed families, cultivars and selections. It allows the exploitation of known pedigree relationships and allows a relatively low marker density [30]. One informative microsatellite marker for every five cM is sufficient for PBA. PBA provides an understanding of the genetic structure of breeding germplasm and discovers the signature of breeders by pinpointing regions that are under high selection pressure without the need for familiarity with the involved traits. Proof-of-concept and statistical analyses of this methodology have been performed in apple through the EU-HiDRAS project [31]. Since then, PBA has been embraced in genetic research on diploid Rosaceae crops [32]. PBA requires advanced statistical software packages, such as FlexQTL™ [17] and Pedimap [18]. MADCE is able to deliver the data required by this software because it can be used to deduce the complete allelic configuration on all homoeologues for a given microsatellite in a given plant, including null alleles and homozygous regions. MADCE thus enables the performance of PBA in complex allopolyploids for the first time. A precursor to PBA was provided by our test set of 21 pedigreed cultivars, in which we were able to follow the flow of marker alleles over pedigrees. MADCE is currently in use for the genotyping of an extensive set of breeding germplasm in strawberries.

### Ability to distinguish disomic from polysomic inheritance

Knowledge regarding the type of polyploidy in a particular plant is critical because the mode of inheritance determines what types of methodology and software can be used in genetic studies. Conventional methods to assess the type of ploidy are based on cytogenetic studies of chromosome pairing behaviour during meiosis [33] and on segregation. The cytogenetic approach cannot provide absolute certainty because multivalents can also be observed in the early stages of meiosis in allopolyploids [34]. In segregation studies, ploidy types are distinguished based on i) segregation ratios for duplex alleles, ii) the occurrence of progeny-genotypes that can only arise due to double reduction, and iii) the ratio by which markers of linked loci are in coupling and repulsion phases [5]. None of these approaches can provide absolute certainty for ploidy type. Segregation ratios cannot provide certainty because it is difficult to distinguish segregation ratios that are greater than 3:1 from each other unless very large populations are used. Additionally, the occurrence of segregation distortion could interfere with these analyses. Double reductions are not reliable because false double reductions can be scored due to phenotyping errors, genotyping errors, outcrossing and DNA admixture. These types of errors generally occur at low frequency, but this is also true for actual double reduction events [35]. Finally, indications through linkage ratios led to the incorrect inference of mixed polysomy and disomy for the cultivated strawberry [13,36]. Moreover, conclusions about the mode of inheritance can only be made after linkage groups have been established, when it is already too late to take advantage of diploid methodology. Finally, this method uses coupling and repulsion phase linkages between loci rather than simply the repulsion allele within a locus. Therefore, as distance increases, the reliability of determining meiotic segregation patterns decreases. In contrast, MADCE offers a very effective approach for establishing the type polyploidy by confirming disomic inheritance prior to mapping through an examination of the presence of allelic pairs within a single locus. If such pairs are found for each of the different chromosomes or a sufficiently large set of random loci, fully functional disomy, and thus allopolyploidy, can be assumed.

### MADCE and new high-throughput genomic tools

Rapid advances in the affordability and throughput of next generation sequencing technologies have sped up the development of high-throughput marker systems. Marker platforms other than microsatellites, including array-based SNP detection technologies [37], fluorescent SNP probes [12,38] and real time quantitative PCR [39], can also be used for dose estimation. These techniques are well developed, accurate and often high-throughput, whereas the use of microsatellites is relatively costly and labour intensive. These platforms are therefore likely to surpass SSR approaches in the near future. However, SNP arrays for allopolyploids are not frequently available because their development is quite complicated. Because sub-genomes are highly differentiated, most SNPs in allopolyploids are likely to be polymorphic between homoeologues but not within a homoeologue. In addition, for the relatively few SNPs that are polymorphic within a homoeologue and can therefore be used in assays, most will have interference from the other sub-genomes. To illustrate this, imagine how well an assay would need to perform to separate the clusters of signals from a SNP assay in an allo-octoploid (AATTTTTT vs. ATTTTTTT vs. TTTTTTTT). This interference would have to be dealt with, for instance by using adjacent sub-genome specific SNPs to make the assays sub-genome specific. The investment required for SNP development is therefore much steeper for higher-order polyploids than for diploids. Other arguments for the continued use of microsatellites is that they are more suitable for application in genetically distinct germplasm due to their high transferability and level of polymorphism. This high level of polymorphism also makes them more likely to tag a haplotype (or trait) than random SNPs. Polyploidy hampers discovery of SNP haplotypes. MADCE could help in the discovery of haplotype tagging SNPs through their association with well-defined microsatellite alleles. Furthermore, information on homozygosity (for cross-pollinators) generated by MADCE enables the selection of the best lines for SNP development by complementing regions that are homozygous and heterozygous between these lines. Based on these perspectives, MADCE currently supports the efforts of the international RosBREED-Illumina consortium in testing SNP development strategies by helping to identify the most appropriate germplasm for use in SNP discovery.

## Conclusions

The MADCE method for the quantitative interpretation of microsatellite data presented in this paper offers a novel tool for the genetic analysis of complex allopolyploid plants. MADCE enhances the genotyping of allopolyploids by dealing with shared alleles between sub-genomes, null-alleles and homozygous loci. This can be used to establish the full allelic configuration of any individual allopolyploid plant. MADCE fosters genetic studies in allopolyploids by increasing the efficiency of generating molecular marker linkage maps and by enabling the fully informative genotyping of pedigreed breeding germplasm. MADCE thus enables the use of statistical and genetic software designed for diploid systems for allopolyploids. MADCE can also be used to aid SNP detection and SNP array development in complex polyploids.

## Methods

### Plant material

For the construction of a molecular marker linkage map, 82 seedlings from a cross between the strawberry cultivars 'Holiday' (H) and 'Korona' (K) were used. For the pedigree analysis, a set of 21 cultivars and breeding lines including 'Holiday' and 'Korona' was used (Figure 4). Leaves were sampled from the Fresh Forward Breeding germplasm collection or made available by the National Clonal Germplasm Repository at Corvallis, Oregon, US.

### DNA isolation

Genomic DNA was extracted according to a modified version of the Fulton et al. [40] mini-prep protocol. Briefly, young, folded leaves were harvested. Leaves were freeze-dried and ground to powder in a 2 ml tube. To this tube, 700 μL of warm (65°C) 2% CTAB buffer was added, and the tube was mixed by vortexing and incubated for 10 min. Next, 700 μL of chloroform:isoamyl alcohol (24:1) was added. The mix was centrifuged at room temperature at 10,000 × g for 2 min. Next, 600 μL of the top phase was transferred to a fresh tube. Isopropanol (480 μL) was added, and the sample was mixed and then centrifuged at 10,000 × g for 2 min at room temperature. The supernatant was discarded, and the pellet was washed with 500 μL of 70% ethanol, left for 2 min and then centrifuged at 10,000 × g for 2 min. The supernatant was discarded by pipetting and the pellet was resuspended in 400 μL of TE. LiCl (135 μL, 8 M) was added to remove RNA and polysaccharides, and the mix was incubated for 30 min at -20°C. After incubation, the mix was centrifuged at room temperature at 10,000 × g for 2 min, and the supernatant was transferred to a fresh tube. Isopropanol (320 μL) was added and the mix was incubated at -20°C for 30 min. The mix was then centrifuged at 10,000 × g for 5 min and the supernatant was discarded. The pellet was then washed and centrifuged twice with 500 μL of ethanol (70%), and then the dried pellet was dissolved in 50 μL of TE.

### Microsatellites

#### Origin

Six microsatellite primer combinations known to be located on a single linkage group (LG1) were taken from a variety of sources (Table 1). Five were used both in the mapping and the pedigree analyses, whereas marker PScontig944 was only used for pedigree analysis.

#### PCR

PCRs were performed with indirect fluorescent labelling with an universal 17 bp 5' end tail sequence (AACAGGTATGACCATGA) on the forward primer that matched a universal fluorescently labelled primer (6-FAM, HEX or ROX). This method was adapted from the protocol described in Schuelke [41]. Reverse primers had a GTTT tail added to them to reduce stutters, according to the protocol from Brownstein et al. [42]. The PCR mix was composed of 1X Goldstar PCR buffer, 0.5 μM unlabelled forward primer with tail, 2 μM of unlabelled reverse primer, 2 μM labelled universal tail primer, 0.3 U of GoldstarTaq polymerase (Eurogentec, The Netherlands) and 10 ng of DNA in a total reaction volume of 20 μL. The PCR conditions were one cycle at 94°C for 3 min, followed by 35 cycles at 94°C for 30 s, 50°C for 30 s and 72°C for 2 min, and a final extension cycle at 72°C for 10 min.

#### Microsatellite data preparation

Depending on amplicon intensity as observed from agarose gel, PCR products were diluted (on average approximately 300× in total) to prevent fluorescent intensity levels to exceed (or be below) the detection levels and thereby hamper dose estimation. Fluorescently labelled amplicons were separated and detected using an ABI capillary automated sequencing platform (ABI 3730, Perkin Elmer Biosystems, Foster City, California). Output from the ABI platform was analysed with Genemapper 4.0 software. Peaks corresponding to alleles were identified and their bin ranges, which are the window of allele sizes that are thought to represent a single allele, were defined. Next, for each sample, the software automatically identified the presence of alleles (peaks), their height and the area under the peak. Proper allele detection was checked manually and adjusted where necessary. The allelic data (size, area) for each individual was transferred to an Excel sheet (see additional files 1, 2, 3, 4, 5). Excel sheets were formatted to show the area data for each individual in rows, and each column represents a different allele. These sheets were then used for further qualitative and quantitative analyses as described in the results section.

### Construction of linkage maps

Linkage maps were created for each parent separately using JoinMap^® ^4.1 (Kyazma B.V., Wageningen, NL) and the Kosambi mapping function. Marker placement was determined using a minimum LOD threshold of 1, a recombination fraction threshold of 0.45, a ripple value of 1 and a jump threshold of 5. Comparisons of the separate parental linkage maps were used for the creation and validation of integrated loci and for error removal. After this data preparation step/upgrade, integrated maps were created using the same JoinMap settings as used for the separate parental maps. Positions for homozygous loci were estimated using interpolation and extrapolation of map distances of the segregating homoeologous loci. Drawings of the linkage maps were first created with the software packages Mapchart [43] and later finalised in Microsoft Powerpoint.

### Pedigree analysis

The 21 cultivars from Figure 4 were genotyped using the six microsatellites in Table 1. Dose and configuration of alleles were established according to the Microsatellite Allele Dose and Configuration Establishment (MADCE) method that was adjusted for pedigreed germplasm, as presented in this paper. Next, the inheritance of these cultivars over a pedigree was analysed using IBD estimates and the most likely linkage phases of alleles according to the software package FlexQTL™ [17]. To graphically represent the flow of alleles over the pedigree, IBD estimates were loaded into Pedimap [18].

## Competing interests

The authors declare that they have no competing interests.

## Authors' contributions

TvD carried out experimental work, analysed the data, contributed to the method development, and drafted the manuscript. TD Contributed to method development. YN Contributed to the experimental work. MB Contributed to data analysis. BM Contributed to the experimental work. RV Contributed to the manuscript. EvdW Conceived project, contributed to method development, and drafted the manuscript. All authors read and approved the final manuscript.

## Supplementary Material

Additional file 1 **ARSFL010 analysis Excel file**. Excel file containing peak area data and analysis of ARSFL010. Click here for file

Additional file 2 **UFFxa16H07 analysis Excel file**. Excel file containing peak area data and analysis of UFFxa16H07. Click here for file

Additional file 3 **UAFv7500 analysis Excel file**. Excel file containing peak area data and analysis of UAFv7500. Click here for file

Additional file 4 **CO817823 analysis Excel file**. Excel file containing peak area data and analysis of CO817823. Click here for file

Additional file 5 **CX661101 analysis Excel file**. Excel file containing peak area data and analysis of CX661101. Click here for file

Additional file 6 **CO817823 analysis MS Word File**. Textual description of the analysis process of CO817823. Click here for file

Additional file 7 **CX661101 analysis MS Word File**. Textual description of the analysis process of CX661101. Click here for file

Additional file 8 **ARSFL010 analysis MS Word File**. Textual description of the analysis process of ARSFL010. Click here for file
