# Supplementary material for: Microsatellite allele dose and configuration establishment (MADCE): an integrated approach for genetic studies in allopolyploids
Source: BMC Plant Biol. 2012 Feb 17;12:25. doi: 10.1186/1471-2229-12-25 (PMC3338383; doi:10.1186/1471-2229-12-25)
Supplement: Additional file 6 — CO817823 analysis MS Word File. Textual description of the analysis process of CO817823. [file 1471-2229-12-25-S6.DOCX]

4. CO817823

The original data for marker CO817823 are presented in the additional file 4. This marker amplifies eight different alleles in total (Table 2 & 3) of which seven segregate for presence and absence. Qualitative analysis of the alleles reveals three alleles with a 1:1 segregation pattern (193H, 209K, 216H) and four alleles with a 3:1 segregation pattern (195, 199, 203 and 236, all HK). For six out of these seven different alleles it is possible to establish allelic pairs qualitatively (193-199 K, 199-203 H, 195-216 H, 195-203 K, 209-236 K), leaving only allele 236H unpaired. The only always present allele, 207HK, shows a 1:1 segregation when using the 1:1 simplex alleles as reference alleles. This makes it impossible to use allele 207 as a reference allele. Because of this, an average based reference is best suited to complete the quantitative analysis. Cursory analysis of peak area’s from single dose alleles indicates large variability in amplification efficiency between the various alleles, thus requiring the use of multipliers in the creation of an average based reference allele. For a perfect virtual reference the allele-areas are multiplied in such a way, that each dose has a similar contribution to the average. For CO817823 (Additional file 4), multipliers are only applied to the alleles with the lowest efficiency (216 and 236). This results in quite good separation between the ratio clusters, so no further optimization is required. Quantitative analysis using this virtual reference confirms the previously found 1:1 dose segregation of allele 207HK. The higher dose of this allele leads to low values or absence of allele 236HK, which allele was already found to be allelic to 209K during the qualitative analysis. Filtering the populations for progenies carrying 209K confirms 207H and 236HK to be allelic: all progenies lacking 236HK have 207HK at high dose and visa versa all seedlings that have 207HK at low dose contain 236HK. As the homoeolog with the segregating 207H allele is complete, the 207Homozyous pair should be on another homoeolog. Allele 207 being present in both parents, this other homoeolog should at least be 207 homozygous for Korona. As for Korona all observed alleles have already been assigned and as Holiday still lacks two alleles, Holiday may either be homozygous 207 or homozygous null. Ratio values of parents and progeny may clarify this. If both parents would be homozygous, Holiday would have three doses, Korona two, and the progeny both three and two. If only Korona would be 207 homozygous, Holiday would have one dose for 207, Korona two, and the progeny both one as well as two doses. Thus, in case of double homozygosity, Holiday is supposed to have ±1.5x higher ratio value of Korona, whereas in case of single homozygosity the ratio value of Holiday is supposed to be half of Korona. The observed ratios match double homozygosity, being 13 for Holiday and 8 for Korona, and suggest a uniform single-dose effect of 4. Next the current model is cross checked through ratio values of parents and progeny. Quantitative analysis of the four 3:1 segregating alleles shows all to exhibit the expected 1:2:1 segregation in RCs except for allele 203. This allele shows a 1:1:1:1 RC segregation for the values 0, 9, 14, 24 respectively. The presence of multiple classes with irregular distances in RC-values indicates the occurrence of amplification efficiency differences, whereby the least and most efficient amplifying allele have a RC-value of 9 and 14 respectively, which fully matches to the parental ratio values (Holiday-14, Korona-9) as well as to the co-segregation patterns of alleles 199H and 195K. The importance of adjusting the calculation of the virtual reference is illustrated by allele 203 for which the uncompensated reference gives a wide distribution for single and double dose clusters (Fig. S1A), and the compensated reference is able to split this single dose cluster into two distinct ditributions as well as narrowing the distribution for double dose (Fig. S1B).

When trying to assign the allelic pairs to the four homoeologs, it turns out that one of the 3:1 segregating alleles needs to be assigned to two homoeologs in order to make the allelic configuration fit to the maximum dose of 8. The most logical solution is to assign the 203 allele to two different homoeologs, because of its efficiency difference. Finally, the sum of all allele doses for this SSR primer combination leads to 8 doses for Holiday as well as for Korona.

Fig. S1A Frequency distribution of ratio values for Allele 203 with uncompensated average.

Fig. S1B Frequency distribution of ratio values for Allele 203 with compensated average.
