# Supplementary material for: Microsatellite allele dose and configuration establishment (MADCE): an integrated approach for genetic studies in allopolyploids
Source: BMC Plant Biol. 2012 Feb 17;12:25. doi: 10.1186/1471-2229-12-25 (PMC3338383; doi:10.1186/1471-2229-12-25)
Supplement: Additional file 7 — CX661101 analysis MS Word File. Textual description of the analysis process of CX661101. [file 1471-2229-12-25-S7.DOCX]

5. CX661101

The original data for marker CX661101 are presented in the additional file 5. Analysis of these data reveals six alleles of which three segregate in presence and absence (Table 2 & 3). These three alleles (220H, 221H and 224K) show a 1:1 segregation and therefore have a simplex configuration. Their repulsion alleles cannot be identified through qualitative analysis. The two 1:1 segregating alleles of Holiday must therefore derive from different homoeologs. Alleles 204H, 212HK, and 223HK are present in the entire progeny. Ratio calculations for 204H with the 1:1 segregating alleles as reference always gives a single cluster, indicating absence of variation in dosage, due to which Holiday should be homozygous for this allele. Allele 212HK is present in both parents as well as in all offspring and has a single RC when examined with simplex alleles. Both parents should thus be homozygous for this allele. Either of these two homozygous alleles can thus be used as a reference allele for the entire progeny. Allele 223HK is present in both parents as well as in the entire progeny indicating homozygosity on at least one genome in at least one of the parents. It appears to segregate in dose, but the distribution of ratio values is not narrow enough to allow complete separation between RCs. This means that as a reference allele 204 and 212 are not of high enough quality to allow complete analysis of allele 223. A ‘virtual’ reference allele is created by averaging the peak areas for each sample. No multipliers have to be used as ratios with single dose alleles indicate amplification efficiencies to be quite uniform. Using this virtual reference we can now clearly distinguish four RCs, indicating the presence of multiple heterozygotes. Allele 223HK must thus be present on at least two homoeologs. The RCs show regular increases (2x, 3x and 4x the lowest ratio of 8) and a 1:3:3:1 segregation (Tables 2 & 3). In disomically inheriting polyploids, this segregation pattern indicates triple heterozygosity. Taking the lowest (8) and highest (31) RCs, corresponding to the lowest (1) and highest (4) allele dose in the progeny, reveals three homologous alleles (220H, 221H and 224K). Consequently, Holiday should have two 1:1 segregating 223-alleles and Korona one. In this way, two segregating allele pairs can be assigned to Holiday and only one to Korona. This makes it likely Korona to be the source for the homozygous 223 pair. Allele configurations thus become 220-223 and 221-223 for Holiday and 223-224 and 223-223 for Korona. Holiday and Korona are therefore assumed to have 223 at double and triple dose respectively, which matches with their dose estimates (Table 3).

For the assignment of the allele pairs to homoeologs, allele doses are summed up per parent: Holiday has the expected eight allele-doses for a marker that amplifies all eight chromosomes of the four homoeologs. However, Korona sums up to only six doses. If we assign all allele pairs to homoeologs (using the shared alleles as a guidance) we can see that the 204 alleles of Holiday have no counterpart in Korona. Combining this information with the presence of only six doses in Korona we can conclude that the homoeolog containing the 204 alleles in Holiday is likely to harbour null alleles in Korona. The current data do not allow conclusions on which of the two Holiday alleles 220H and 221H are homologous to 224K. Conclusions on this require co-segregation (mapping) data with linked markers or transmittance data.
