# Supplementary material for: Microsatellite allele dose and configuration establishment (MADCE): an integrated approach for genetic studies in allopolyploids
Source: BMC Plant Biol. 2012 Feb 17;12:25. doi: 10.1186/1471-2229-12-25 (PMC3338383; doi:10.1186/1471-2229-12-25)
Supplement: Additional file 8 — ARSFL010 analysis MS Word File. Textual description of the analysis process of ARSFL010. [file 1471-2229-12-25-S8.DOCX]

*Microsatellite ARSFL010*

Marker ARSFL010 amplifies six different alleles, and five of these segregate for presence and absence (Table 2 & 3, additional file 1). H is used to indicate an allele from ‘Holiday’, K indicates an allele from ‘Korona’, and HK denotes that the allele is present in both parents. Qualitative analysis reveals that three maternal alleles, 234H, 248H and 257H, and two paternal alleles, 246K and 286K, inherit in a simple 1:1 presence/absence pattern. Two allelic pairs can be identified by qualitative analysis (234H-257H and 246K-286K). For allele 269HK, which is always present, the area ratios of each of the single dose alleles that are used as references give a single RC each time, which indicates the absence of segregation. Allele 269HK must therefore be homozygous in both parents. This allele is a good reference allele because its size is different from any other allele and it does not have stutter bands. For 248H, no homologous allele is found, which implies the existence of a null allele. Consequently, for ‘Holiday’, three homologous pairs are identified: 269H-269H, 234H-257H and 248H-0H. For ‘Korona’, the 269K-269K and 246K-286K pairs areidentified. Next, we examine whether amplification intensities or peak shape could assist in the matching between the 246-286 homoeologue of ‘Korona’ to one of the ‘Holiday’ homoeologues. Ratio analyses using 269 as a reference show that 246K and 257H have similar high amplification ratios, whereas 248H shows a much lower value (16, 15 and 4, respectively). Consequently, allele pairs 246K-286K and 234H-257H are likely to be homologous. The low ratio of the 248H allele can be potentially explained by primer site mutations, which would lead to poor amplification. Because there are no un-assigned alleles, the 248H-containing homoeologue is concluded to contain 3 null alleles. This leaves us with a fourth homoeologous set, which could either contain four null alleles or four 269 alleles. The peak area of 269 is smaller than the peak area of the 257 simplex allele (Additional file 1). This makes it unlikely that the 269 allele is homozygous at two homoeologues for both parents because that would imply that a quadruplex dose of 269 has a smaller peak area than a simplex dose of 257. Additionally, it was previously established that null alleles are present on one homoeologue, which adds to the possibility of them also occurring at other homoeologues. Therefore, we conclude that the fourth homoeologue probably contains only null alleles for both parents. The occurrence of allele 269 remains restricted to one homoeologue.
